# Reply to “Missed opportunities in large scale comparison of QSAR and conformal prediction methods and their applications in drug discovery”

**DOI:** 10.1186/s13321-019-0388-x

**Published:** 2019-11-06

**Authors:** Nicolas Bosc, Francis Atkinson, Eloy Félix, Anna Gaulton, Anne Hersey, Andrew R. Leach

**Affiliations:** Chemogenomics Team, European Bioinformatics Institute (EMBL-EBI), Wellcome Genome Campus, Hinxton, Cambridge, CB10 1SD UK

## Abstract

In response to Krstajic’s letter to the editor concerning our published paper, we here take the opportunity to reply, to re-iterate that no errors in our work were identified, to provide further details, and to re-emphasise the outputs of our study. Moreover, we highlight that all of the data are freely available for the wider scientific community (including the aforementioned correspondent) to undertake follow-on studies and comparisons.

## Introduction and background

Krstajic recently published a letter to the editor [1] concerning our paper [2]. In this work we described an extensive investigation into the application of Mondrian Conformal Prediction (MCP) methods to the creation and application of in silico “target prediction” models, which enable the activity of a compound against a particular biological target protein to be predicted and a confidence level to be assigned to that prediction. The methodology behind this technique is described in our article [2], and the interested reader may find a recent review useful [3]. Our work involved the creation of 550 MCP models, using data from the ChEMBL database [4], and a series of detailed and in-depth analyses. We also described the practical applications of such models with a number of specific illustrative examples. Because it is still a relatively novel technique, we decided to compare the results obtained with MCP at different confidence levels with those delivered with a more classic QSAR approach to see how the two differ.

Our original paper was reviewed by multiple anonymous referees, whose detailed comments and feedback indicated that they were experts not only in QSAR but also in conformal prediction. We addressed their comments and suggestions in detail, resulting in the paper that was eventually published. Following publication, we had a number of email exchanges with Krstajic in which we explained and expanded in detail on the work in our paper and also provided background information on the underlying theory and practical applications of conformal prediction. These exchanges formed the basis of the subsequent letter [1]. Here, we therefore take advantage of the opportunity afforded us by the editor to provide our responses in public, which we do in some detail below. First, however, we wish to make a more general point. This is that there is a significant degree of subjectivity in how one chooses to implement QSAR and MCP methods, and that we consider our published implementation to be entirely consistent with current best practice in the field, as outlined in [3]. We further note that our paper has been viewed or downloaded at least 2600 times according to the journal’s website and [1] represents the only adverse comments that we have received to date.

## In-depth comments

In this section we address the specific criticisms made in [1]:

- The extent to which our comparison between QSAR and MCP can be generalised
- The variability of our results
- The validation of our models

Throughout his letter, the author variously agrees and disagrees with our approach. A number of his criticisms arise from what we would consider an overly literal interpretation of our wording. A simple example is our choice of title, and the implication that we are making broader and more significant claims than are justified (“… in our opinion that cannot be the reason, nor do the authors provide any explanation, for generalising their findings in the paper (most importantly in the title, abstract, graphs, tables and conclusion) as QSAR vs MCP models”). Whilst we accept that a literal interpretation of the title of our paper could be misleading, we would counter that we are simply following convention and precedent and that throughout our paper we have been very clear on the methods we have used, their scope and limitations. A significant number of previous publications use the terms QSAR or Conformal Prediction (CP) in their titles without indicating which machine learning method was used. Indeed, of 28 articles described in [3], 18 use Random Forests exclusively or with other methods without mentioning RF in the title. Our Methods section contains the full details of the approaches we employed.

Krstajic declares that: “When performing a comparison between two methods, in our view, it is very important to address the issue of the variability of generated results”, with specific mention of (a) the train/calibration/test splitting mechanism, (b) the use of random number seeds and (c) the representation of error bars. For each of the 100 model-building iterations the random seed that determines the constitution of the train/validation/test sets was pre-allocated. We further used the exact same set of random forest parameters at each iteration. This approach was chosen to enable us and others to reproduce exactly our work, which was one of our main drivers. However, we acknowledge that for each of the 100 iterations we explored just one train/calibration split for MCP. It is therefore possible that there may be some small additional variability in the overall results due to this cause, though we would anticipate it to be extremely limited (and probably not detectable). We are also happy to clarify that the errors bars in Figs. 3 and 4, and the ± values indicated in the text or in the tables all correspond to the standard deviation over the relevant population, consistent with standard practice [5, 6].

Concerning our approach to model validation, for each of the 100 iterations we took each compound in the test set and derived the prediction. Then, in order to derive a prediction for each compound across all 100 iterations we determined the median probability (for QSAR) or the p value (for MCP). Of course, due to the “random” nature of the distribution of compounds in the 100 training sets, the actual number of predictions per compound will vary (in theory, from 0 to 100, with an average of 20 due to the 80:20 split used for training and testing). We therefore confirm Krstajic’s statements concerning this, but again we believe that our conclusions, which are drawn across 550 models, each of which represents 100 iterations, are sound.

## Conclusions

We appreciate the author’s efforts to scrutinise our experiments. We also acknowledge that perhaps some additional information may have been useful in understanding the details of each step. He has presented some interesting ideas on potential future work that would build on our published studies. Our datasets have been available since the publication date of our paper (at http://ebi.ac.uk/pub/databases/chembl/qsar_vs_cp_modelling_data); we would encourage Krstajic to undertake these additional analyses and to publish his results.

## Data Availability

Not applicable.

## Abbreviations

CP: Conformal Prediction
MCP: Mondrian Conformal Prediction
QSAR: Quantitative Structure Activity Relationship

## References

[1] Krstajic D (2019). Missed opportunities in large scale comparison of QSAR and conformal prediction methods and their applications in drug discovery. J Cheminform.

[2] Bosc N, Atkinson F, Felix E (2019). Large scale comparison of QSAR and conformal prediction methods and their applications in drug discovery. J Cheminform.

[3] Cortés-Ciriano I, Bender A (2019) Concepts and applications of conformal prediction in computational drug discovery. arXiv:190803569 [cs, q-bio]

[4] Mendez D, Gaulton A, Bento AP (2019). ChEMBL: towards direct deposition of bioassay data. Nucleic Acids Res.

[5] Cortés-Ciriano I, Firth NC, Bender A, Watson O (2018). Discovering highly potent molecules from an initial set of inactives using iterative screening. J Chem Inf Model.

[6] Cortés-Ciriano I, Bender A (2019). KekuleScope: prediction of cancer cell line sensitivity and compound potency using convolutional neural networks trained on compound images. J Cheminform.

